# Large-Scale Discovery of Disease-Disease and Disease-Gene Associations

**DOI:** 10.1038/srep32404

**Published:** 2016-08-31

**Authors:** Djordje Gligorijevic, Jelena Stojanovic, Nemanja Djuric, Vladan Radosavljevic, Mihajlo Grbovic, Rob J. Kulathinal, Zoran Obradovic

**Affiliations:** 1 Center for Data Analytics and Biomedical Informatics, Temple University, Philadelphia, PA 19122 USA; 2 Department of Biology, Temple University, Philadelphia, PA 19122 USA; 3 Institute of Genomic and Evolutionary Medicine, Temple University, Philadelphia, PA 19122 USA

## Abstract

Data-driven phenotype analyses on Electronic Health Record (EHR) data have recently drawn benefits across many areas of clinical practice, uncovering new links in the medical sciences that can potentially affect the well-being of millions of patients. In this paper, EHR data is used to discover novel relationships between diseases by studying their comorbidities (co-occurrences in patients). A novel embedding model is designed to extract knowledge from disease comorbidities by learning from a large-scale EHR database comprising more than 35 million inpatient cases spanning nearly a decade, revealing significant improvements on disease phenotyping over current computational approaches. In addition, the use of the proposed methodology is extended to discover novel disease-gene associations by including valuable domain knowledge from genome-wide association studies. To evaluate our approach, its effectiveness is compared against a held-out set where, again, it revealed very compelling results. For selected diseases, we further identify candidate gene lists for which disease-gene associations were not studied previously. Thus, our approach provides biomedical researchers with new tools to filter genes of interest, thus, reducing costly lab studies.

The increased penetration of information technologies in hospital systems in recent years has enabled collections of vast amounts of medical data in the form of *electronic health records* (EHRs). EHRs contain detailed patient-related data collected over time including past medical history, medications, procedures, immunizations, and diagnostic findings. In addition, EHRs store information concerning all stages of inpatient care, including a *patient discharge summary*, a detailed report prepared by a clinician at the end of each hospital stay. This document also contains a comprehensive list of patient’s diagnostic findings, as well as the administered procedures. Clearly, such a rich source of patient-specific data presents an unprecedented opportunity to apply data-driven approaches for knowledge discovery in clinical research^1^.

Data mining researchers have recognized the value and potential of inpatient medical data, and have recently proposed effective mining approaches to help obtain actionable insights for improving healthcare^2^. However, the modeling process is burdened by a number of challenges, as the data often contains sparse, heterogeneous, and incomplete information due to different hospital and insurance polices, further aggravated by non-standardized physician practices^3^. The existing tools are not fully capable of addressing such a challenging task^4^, and in order to make use of these multifaceted noisy data, development of novel machine learning approaches is required to allow for efficient and effective analysis. Additionally, a vast amount of medical knowledge is available, even though often incomplete^5^,^6^, that could be used to improve the power of these models^7^,^8^. Examples of such sources are disease and gene ontologies, protein-protein interactions, and discovered disease-gene associations from previous medical studies. Building models capable of including such available domain knowledge could dually improve over original approaches: first, domain knowledge can increase performance of the original models, and second it can allow for novel applications and discoveries not possible before.

In this paper, a novel route is proposed for disease phenotyping and gene discovery, a critical step in the deeper understanding of medical conditions and drug discovery^9^. This work is motivated by recent advances in the field of natural language processing (NLP)^10^,^11^, and is capable of seamlessly addressing the inherent issues of sparsity and heterogeneity present in medical data records. In particular, a distributed, neural embedding model is proposed for the phenotypic discovery of diseases that often co-occur in patients (referred to as *disease comorbidity*), and are expected to be governed by the same genetic mutations^12^. Our proposed approach is further developed to allow inclusion of domain knowledge in terms of previously discovered disease-gene associations, improving over original approach on the disease phenotyping task formulated as an information retrieval task and allowing for discoveries of previously unknown disease-gene associations. The goal of disease phenotyping task considered in our study is to examine which representation is genetically the most relevant, when genes were not included in the model training and where a hold-out set is used for evaluation. The parallel can be made with document retrieval studies where *k* nearest documents are retrieved and success is evaluated by a similarity metric. In such experiments our proposed approach is shown to be more accurate than other state-of-the-art approaches with respect to a number of rigorous evaluation tasks. A summary of the proposed approach is illustrated in Fig. 1.

We summarize the contributions of this work below:

- An application of distributed language models is proposed for the phenotypic discovery of disease associations. A novel method is used for learning low-dimensional disease representations that compactly capture their relations.
- A framework is proposed for inclusion of domain knowledge in the learning process. Specifically, gene association information is incorporated into EHR patient discharge data, which allows for learning low-dimensional gene and disease representations in the same vector space, as well as for the discovery of novel gene-disease interactions through straightforward nearest-neighbor searches.
- We trained and evaluated our models using large-scale EHR data comprising more than 35 million patient records, resulting in a model of high quality. The results on the task of disease phenotyping show that the proposed method achieved up to 85.98% accuracy, outperforming state-of-the-art methods by a very large margin.
- Genetic associations from GWAS studies also provide independent evidence that the proposed method is capable of discovering genetically meaningful phenotypes from noisy EHR data. To further examine the quality of our discovered phenotypes, use-case analysis is conducted for several disease phenotypes providing evidence of meaningful medical discoveries.
- The use of the proposed methodology is extended to the task of disease-gene relationship discovery. To evaluate the value and potential of the proposed approach, its effectiveness is compared to state-of-the-art methods and evaluated on a held-out set. For example, in the case study of Congestive Heart Failure (CHF), 185 genes were retrieved using our method out of the 185 GWAS-derived genes associated with CHF.
- To facilitate further developments in the field and to follow-up investigations by biomedical researchers, we provide candidate gene lists of disease-gene associations that were not previously studied.

The following section reviews existing approaches for disease phenotyping and is followed by a section where a novel approach for this task is proposed. Extensive evaluation results of the proposed approach on tasks of disease phenotyping and gene discovery, as well as descriptions of datasets used in this study, are given a later section. Finally, we provide conclusions and discuss future work.

## Background and related work

In the treatment of ailments, the focus of medical practitioners can be roughly divided between two complementary approaches: 1) treating the symptoms of already sick patients (reactive medicine); and 2) understanding disease etiology in order to prevent manifestation and further spread of the disease (preventative medicine). In the first approach, the disease symptoms are a part of a broader phenotype profile of an individual, with *phenotype* being defined as the presence of a specific observable characteristic in an organism, such as blood type, response to administered medication, or the presence of a disease^13^. The identification process of useful, meaningful medical characteristics and insights for the purposes of medical treatment is referred to as *phenotyping*^14^. In the second approach, researchers identify the genetic basis of disease by discovering the relationship between exhibited phenotypes and the patient’s genetic makeup in a process refereed to as *genotyping*^15^. Establishing a relationship between a phenotype and its associated genes is a major component of *gene discovery* and allows biomedical scientists to gain a deeper understanding of the condition and a potential cure at its very origin^16^. Gene discovery is a central problem in a number of published disease-gene association studies, and its prevalence in the scientific community is increasing steadily as novel discoveries lead to improved medical care. For example, results in the existing literature show that gene discovery allows clinicians to better understand the severity of patients symptoms^17^, to anticipate onset and path of disease progressions (particularly important for cancer patients in later stages^18^), or to better understand disease processes on a molecular level enabling the development of better treatments^19^. As suggested in previous studies^20^, such knowledge may be hidden in vast EHR databases that are yet to be exploited to their fullest potential. Clearly, both phenotyping and gene discovery are important steps in the fight for global health, and advancing tools for these tasks is a critical part of this battle. The emerging use of gene editing techniques to precisely target disease genes^21^ will require such computational tools at precision medicine’s disposal.

EHR records, containing abundant information relating to patients’ phenotypes that have been generated from actual clinical observations and physician-patient interactions, present an unprecedented resource and testbed to apply novel phenotyping approaches. Moreover, the data is complemented by large amounts of gene-disease associations derived from readily available genome-wide association studies. However, current approaches for phenotyping and gene discovery using EHR data rely on highly supervised rule-based or heuristic-based methods, which require manual labor and often a consensus of medical experts^22^. This severely limits the scalability and effectiveness of the process^3^. Some researchers proposed to combat this issue by employing active learning approaches to obtain limited number of expert labels used by supervised methods^23^,^24^. Nevertheless, the state-of-the-art is far from optimal as the labeling process can still be tedious, and models require large numbers of labels to achieve satisfactory performance on noisy EHR data^3^. Therefore, we approach solving this problem in an unsupervised manner.

Early work on exploiting EHR databases to understand human disease focused on graphical representations of diseases, genes, and proteins. Disease networks were proposed in Goh *et al*.^25^ where certain genes play a central role in the human disease interactome, which is defined as all interactions (connections) of diseases, genes, and proteins discovered on humans. Follow up studies by Hidalgo *et al*.^26^ proposed human phenotypic networks (commonly referred to as comorbidity networks) to map with disease networks derived from EHR datasets, which were shown to successfully associate a higher connectivity of diseases with higher mortality. Based on these advances, a body of work linked predictions of disease-disease and disease-gene networks^6^,^27^ even when a mediocre degree of correlation (~40%, also confirmed on data used in this study) was detected between disease and gene networks, indicating potential causality between them. Such studies provided important evidence of modeling disease and human interactome networks to discover associated phenotypes. Recently, network studies of the human interactome have focused on uncovering patterns^28^ and, as the human interactome is incomplete, discovering novel relationships^5^. However, it has been suggested that network-based approaches to phenotyping and discoveries of meaningful concepts in medicine have yet to be fully exploited and tested^29^. This study offers a novel approach to represent diseases and genes by utilizing the same sources of data as network approaches, but in a different manner, as discussed in greater detail in the section, below.

In addition, to create more scalable, effective tools, recent approaches distinct from networks have focused on the development of data-driven phenotyping with minimal manual input and rigorous evaluation procedures^3^,^30^,^31^. Part of the emerging field of *computational phenotyping* includes the methods of Zhou *et al*.^32^ which formulates EHRs as temporal matrices of medical events for each patient, and proposes an optimization-based technology for discovering temporal patterns of medical events as phenotypes. Further, Ho *et al*.^33^ formulated patient EHRs as tensors, where each dimension is represented by a different medical event, and the use of non-negative tensor factorization in the identification of phenotypes. Deep learning has also been applied to the task of phenotyping^30^, as well as graph mining^31^ and clustering^34^, used to identify patient subgroups based on individual clinical markers. Finally, Žitnik *et al*.^35^, conducted a study on non-negative matrix factorization techniques for fusing various molecular data to uncover disease-disease associations and show that available domain knowledge can help reconstruct known and obtain novel associations. Nonetheless, the need for a comprehensive procedure to obtain manually labeled samples remains one of the main limitations of modern phenotyping tools^14^. Although state-of-the-art machine learning methods have been utilized to automate the process, current approaches still observe degraded performance in the face of limited availability of labeled samples that are manually annotated by medical experts^36^.

In this paper, we compare representatives of the above approaches against our proposed approach in a fair setup and, overall, demonstrate the benefits of our neural embedding approach (described below) on several tasks in a quantifiable manner.

## The proposed approach

To address the shortcomings of the existing state-of-the-art methods for disease phenotyping, we propose a radically new approach, motivated by the recent success of distributed language models in Natural Language Processing (short NLP) applications^11^,^37^. In the context of NLP, distributed models are able to learn useful word representations in low-dimensional continuous vector spaces in an unsupervised manner, without the need for expensive labeling/annotation efforts. The methods use the surrounding context of a word in a sentence, and learn word representations such that in the resulting embedding space semantically similar words are close to each other^11^. Our objective is to take advantage of this property for the task of disease phenotyping, and learn disease representations in a low-dimensional space where diseases that occur in the same contexts are nearby. As a result, and in contrast to comorbidity methods commonly used in practice, related diseases could have a high similarity score even if they do not co-occur in the same patients. This would allow identification of similar diseases through straightforward *K*-nearest-neighbor search in the disease embedding space, without using supervised signals during the learning process. A similar approach has been successfully applied to extracting features from medical texts^38^. However, adopting such an approach to extract meaningful concepts from EHR databases coupled with other heterogeneous sources, as proposed in our study, is the first work of its kind.

Adapting distributed language models to the task of disease phenotyping is not an easy endeavor. Finding distributed disease representation, as opposed to finding word representations, brings very unique challenges quite different from those found in everyday NLP problems. Contrary to everyday language where linguistic rules and notions of words and sentences are clearly defined, there are no existing notions of “sentence of diseases” or surrounding contexts that are equivalent to the NLP domain.

In this paper, we address these issues, and propose two methods that bring state-of-the-art distributed language models to the setting of disease phenotyping: 1) *Disease2Diseases*, where we exploit inpatient discharge summaries from EHR records, from which we create “disease sentences” and apply recently proposed language model^11^; and 2) *DiseaseAndGenes2Diseases*, where we propose a novel method to learn disease and gene vector representations simultaneously by incorporating domain knowledge regarding known disease-gene associations into the inpatient discharge observational data. The *DiseaseAndGenes2Diseases* method learns low-dimensional representations of diseases and genes in the same embedding space^10^, which opens doors for application of the proposed method to a number of important tasks, such as the discovery of new disease-gene associations.

### Low-dimensional embedding models

Let us assume that we are given a set 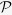 of patient discharge records and a set 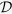 of possible diseases. Then, a discharge record 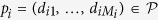 of the *i*^th^ patient is defined as a sequence of diseases 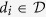 at the end of a hospital stay, where *M*_*i*_ is the number of diagnosed diseases in the sequence. Moreover, each disease 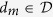 is associated with *N*_*m*_ genes, called a genotype of the disease, represented as a sequence of genes 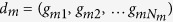, 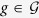, where 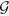 is the set of all possible genes. Then, using the set 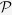, the objective is to find *D*-dimensional real-valued representations 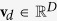 for every disease *d* and 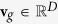 for every gene *g*, such that diseases with similar phenotypes and common gene origins lie nearby in the vector space.

Before discussing their application to disease phenotyping, let us introduce the main idea of neural language models as applied to NLP. These methods take advantage of word order, and state the same assumption as *n*-gram language models that temporally closer words in the word sequence are statistically more dependent. Typically, a neural language model learns the probability distribution of the next word given a fixed number of preceding words that act as the context. More formally, given a word sequence 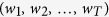 from the training data, the objective of the model is to maximize the average log-likelihood function,


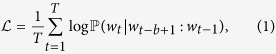
where *w*_*t*_ is the *t*^th^ word, and 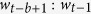 is a sequence of *b* successive preceding words that act as the context to the word *w*_*t*_. A typical approach to approximate probability distribution 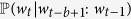 is to use a neural network model architecture^39^. The neural network is trained by projecting the vectors for context words 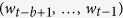 into a latent representation with multiple non-linear hidden layers and the output softmax layer comprising *W* nodes, where *W* is a size of the vocabulary (equal to the number of diseases 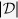 in our task), while attempting to predict word *w*_*t*_ with high probability.

When working with large-scale data, the vocabulary size *W* can easily reach the millions. In those cases, training of the neural network becomes a challenging task, as updates of word vectors become computationally expensive. For that reason, recent approaches^11^ propose log-linear models which aim to reduce the computational complexity. The use of hierarchical softmax^40^ or negative sampling^11^ is shown to be effective in substantially speeding up the training.

### Disease2Diseases (D2D) method

In this section we propose the *disease2diseases (D2D)* approach for learning disease representations, building upon ideas introduced by the recently proposed *word2vec* algorithm^11^. The key insight is that we can represent the patients’ lists of diseases and medical conditions from EHRs as sequences of tokens, and view each sequence as a sample from some unknown language. Following this reasoning, the language model learns representations of diseases in a low-dimensional space using each patient discharge record as a “sentence” and the diseases within the record as “words”, to borrow the terminology from the NLP domain. The diseases in each record are ordered by the time of their diagnosis, from earlier to more recently found conditions. Low-dimensional disease representations are learned by maximizing the objective function 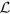 over the entire set 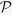 of records as follows,


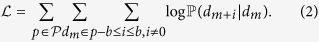
The probability 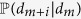 of observing some “neighboring” disease *d*_*m*+*i*_ given the current disease *d*_*m*_ is defined using the soft-max function as


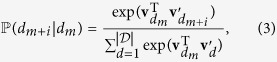
where **v**_*d*_ and 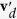 are the input and output *D*-dimensional vector representations of disease *d*, and hyper-parameter *b* represents the length of the context for disease records.

**Figure 2:**
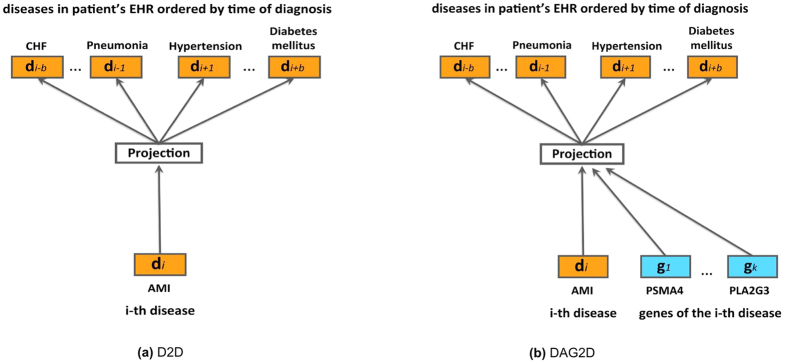
Graphical representations of the D2D and DAG2D models illustrated on projecting Acute Myocardial Infarction (AMI) diagnoses and AMI-related genes to AMI-associated diagnoses.

As illustrated in Fig. 2(a), and equation (3), *Disease2Diseases* uses the central disease *d*_*m*_ to predict *b* diseases that come before and *b* diseases that come after it in the discharge record. As a result, diseases that often co-occur and diseases with similar contexts (i.e., with similar neighboring diseases) will have similar representations as learned by our model.

### DiseaseAndGenes2Diseases (DAG2D) method

In the previous section we described how we can learn disease representations directly from EHR records in an unsupervised manner. However, there exists a large amount of domain knowledge related to the observed diseases, and omitting this valuable information during modeling and training stages would clearly lead to suboptimal performance of any approach^41^. In this section we describe *DiseaseAndGenes2Diseases (DAG2D)*, a method that allows straightforward incorporation of external information into the training procedure, resulting in improved vector embeddings.

The DAG2D model assumes that a subset of diseases from the training data 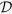 are associated with genes, where the associations are provided by domain experts and considered as domain knowledge. We leverage this information by assigning a vector representation to each gene, and make use of disease contexts in the training data to jointly learn both disease vectors and gene vectors in the same low-dimensional space. To this end, given the diseases associated with genes, we extend the set of patient discharge records 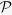 to obtain data set 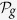, where associated genes were added to the discharge records. In particular, assuming that a disease in the EHR record is accompanied by a non-empty set of associated genes, whenever a vector of central disease *d*_*m*_ is updated to predict the surrounding diseases, the vectors of genes assigned to *d*_*m*_ are updated as well.

More formally, assuming central disease *d*_*m*_ is associated with *N*_*m*_ of 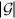 genes in total, 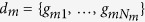, the DAG2D learns disease and gene representations by maximizing the following objective function 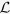,


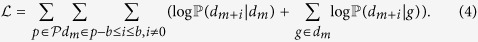
Probability 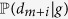 of observing neighboring disease *d*_*m*+*i*_, given gene *g* associated with the central disease *d*_*m*_, is defined using the soft-max,


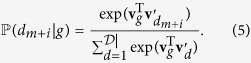


The DAG2D model is depicted in Fig. 2(b), where we illustrate how the context disease vectors are influenced both by the central disease and by its associated genes.

We solve both (2) and (4) using stochastic gradient descent, suitable for large-scale problems. However, computation of gradients is proportional to the number of unique diseases and genes in the data, which may be computationally expensive in practical tasks. As an alternative, we use negative sampling^11^, which significantly reduces the computational complexity and allows fast training of the embeddings on data with millions of patient records. Lastly, once the disease and gene vectors are trained, we measure similarity between them using the cosine distance.

Both D2D and DAG2D models can be seen as weighted matrix factorization models of underlying disease context structure^42^. This neural embedding approach can be compared to other matrix factorization models on different disease network and covariance matrices, with the advantage of being better able to explore disease co-occurence^42^.

The proposed approach has certain drawbacks in terms of modeling. For instance, parameters *D* and *b* are not automatically selected. Additionally, each disease in this study receives a single vector representation, whereas, in reality, the same disease can have several modules: for example sepsis caused by the pneumonia and sepsis caused by external injury. Also, the current model does not take into account disease hierarchical structure which can carry significant information. These issues will be addressed as a follow up: the main goal of this study is to characterize the power of disease representations of the proposed neural embedding models.

## Experiments

In this section we describe the data sets used to evaluate the proposed embedding methods, introduce baseline methods, and discuss the experimental setup and evaluation results.

### Data sets

The primary data source used in this paper is from the public State Inpatient Database^43^ (SID), a set of longitudinal state-specific hospital inpatient databases. This rich dataset is provided by the Agency for Healthcare Research and Quality, and is a part of the Healthcare Cost and Utilization Project (HCUP). Specifically, we collected EHR data from SID California, containing 35,844,800 discharge records from 474 hospitals over a period of 9 years (from January 2003 to December 2011). For each patient there are up to 25 diagnosis codes, originating from the 9th revision of the International Classification of Diseases (ICD9), a hierarchical coding scheme which is part of standard diagnostic tools for epidemiology, health management, and clinical practice^44^. The ordering of diagnoses is used as found in the database (diagnoses codes are ordered by the importance for inpatient admission as seen in doctors’ notes at the time of discharge), given that it is built by ranking diagnoses from the doctors’ notes for each patient; thus the first listed disease is the primary reason for hospitalization, with secondary diseases diagnosed at admission or during hospitalization. In our experiments, we limit the population to inpatients who are more than 1 year old. In total, the SID California database includes 14,207 unique disease codes.

In addition to observational EHR data obtained from hospitals, we used domain knowledge data that contains genetic variations associated with a particular disease, collected from published results of various medical studies. In particular, we used the EBI-NHGRI public GWAS catalog data^45^, which contains disease-gene associations for more than 11,000 genes and 71 disease groups (out of 260 disease groups defined in the ICD9 Clinical Classifications Software schema). In order to create a unified mapping between genes and diseases, we map a gene to a disease group using single nucleotide polymorphisms (SNPs) with a p-value < 10^−5^. The ordering of genes for each disease is possible using p-values, however, as studies are conducted on different human populations, such ordering could be potentially biased. Therefore, we shuffle genes for each disease at each different discharge record in our experiments to ensure the removal of this bias provided by the studies. In addition to hand curating the GWAS database, we have also manually introduced gene-disease mappings from PubMed publications.

Note that there are around 190 disease groups for which no gene associations were previously investigated (e.g., thyroid disorders). In order to improve the understanding of these understudied diseases, medical researchers can greatly benefit from our methods that suggest potential gene associations. Concentrating on a subset of suggested genes would significantly reduce time and monetary costs needed for research studies.

### Experimental setup

To demonstrate the power of the models, we evaluate them on two tasks:

- *Disease Phenotyping*: Identifying diseases with similar contexts as the query disease.
- *Disease-Genes Association Discovery*: Identifying (novel) disease-genes relations of the query disease.

For the first task, the models are trained in two set-ups. To train the D2D model, we used only EHR data (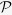 dataset). To train the DAG2D model (for both of the tasks), we extended patient discharge records by associating diseases to corresponding genes according to GWAS data (we found 2,739 diseases that have gene associations, or 23.5% of the entire disease set) in 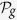 dataset. In order to remove any bias across studies, gene lists are shuffled while assigning the list to the disease.

Dimensionality of the embedding space *D* was explored for 11 choices in the 50 to 1,000 range. Context neighborhood size was set to *b* = 8 chosen to be close to average number of diagnosis of all inpatient records (7.58 in our dataset). Finally, we used 25 negative samples in each vector update for negative sampling as suggested in the literature^11^. Following reported distributed language models^11^, the most frequent diseases and genes were subsampled during training.

For evaluation and comparison with the current state-of-the-art we pose the tasks of phenotyping and gene discovery as information retrieval tasks. First, the proposed model and baselines are used to learn disease and gene representations in an unsupervised manner. Then, for each disease we retrieve k-nearest diseases or genes in the embedded space, and evaluate the quality of the retrieved objects using *Precision@K* metric.

**Figure 3:**
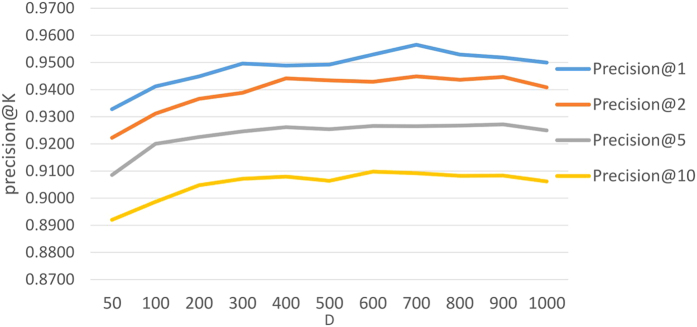
*Precision@K* for D2D model with different dimension *D* of the embedding space.

Embedded dimensionality *D* was chosen to be smallest *D* where *Precision*@*K* starts saturating as dimensionality grows. In our experiments, this point was observed at dimensionality *D* = 200, which provided an acceptable trade-off between good accuracy of the model and its training speed which scales linearly with the dimensionality (Fig. 3). While increasing *D*, we have observed drop in accuracy and halted further examinations of dimensionality in this study.

#### Baseline models set-up

We evaluated the proposed methods against state-of-the-art approaches, such as *1)* Latent Dirichlet Allocation (LDA)^46^, *2)* spectral clustering^47^, and *3)* modularity^48^, which have been successfully applied to EHR analysis^49^,^50^. The LDA model was trained using the same data as D2D and DAG2D. The spectral and modularity models representation in *R*^*d*^ from the first d eigenvectors were trained by decomposing the Laplacian of the graph *G* and modularity matrix of graph *G*, respectively. We define two types of graph *G* in which:

- Nodes represent diseases and genes, and links are determined by gene co-occurrence in GWAS and disease co-occurrence in EHR data.
- Nodes represent diseases, and links are determined by the comorbidities in the EHR data as proposed in Hidalgo *et al*.^26^. For each link, a Pearson correlation is defined, and link rejection decided using a t-statistic^6^ (disease-gene network was not built using comorbidities statistics, as such an approach is not used in the literature).

It should be noted that there are other ways to generate interactome networks of human diseases^5^,^27^,^28^,^51^,^52^, however, these are not easily applicable for a general disease phenotyping task this study addresses, and as such are not included.

The diseases and genes are then mapped into a 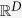 space by projecting onto the subspace spanned by the largest eigenvectors. In order to compare to the largest body of work on disease representation, we have drawn disease phenotypes by choosing the nearest neighbors (the largest link weight) of the query disease in the *4)* disease comorbidity network, as well as in the *5)* diseases and diseases-genes co-occurance network. The *5)* can be seen as a baseline that for a particular disease returns neighbors that were most frequently commonly observed in the EHR data. In addition, disease comorbidity representation was calculated by applying random walks on the comorbidity network^53^, however this approach failed to provide satisfactory results due to graph sparsity, as such, those results are omitted from the Results section.

### D2D based disease-disease associations

In each approach we map diseases to *D* = 200 dimensional space. Then, disease-disease closeness values are measured in the embedded space using the cosine distance metric.

In the first set of experiments we evaluated the quality of disease representations obtained using the two proposed methods. Specifically, we selected 2,739 diseases found in the GWAS data and for each retrieved *K* nearest diseases, with 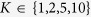. Each of the retrieved diseases was labeled as positive if it shares a gene with the query disease, and labeled negative otherwise, which is used as a proxy for having the same phenotype^54^. Then, we computed *precision@K* for each disease as a fraction of positive neighbors within the *K* retrieved ones, and report the average precision over all 2,739 diseases in Table 1.


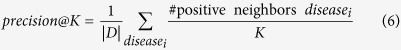
Our proposed methods outperform other approaches by a significant margin, for all values of *K* and for both training data sets, suggesting the suitability of the approaches for the task of phenotyping.

Moreover, as each disease has more than one gene associated with it in the GWAS data, we computed an overlap of the genes between the query disease and its neighbor. Then, for each query disease we computed the percentage of overlapping genes^6^ as


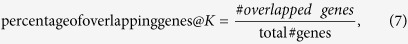
giving a stronger measure of genetic similarity between the neighboring diseases. We report the average overlap over all diseases in the right side of Table 1. Again, based on the reported results we find that our proposed approaches obtained the best results, providing much better disease representations than any of the state-of-the-art methods.

### Case studies of D2D-based retrieval of disease-disease associations

To illustrate the usefulness of the D2D model we discuss disease-disease associations discovered by this approach in the context of four specific high-impact diseases. Case studies demonstrate the power of improved disease phenotyping in increasing clinical knowledge by both generating novel association discoveries and decreasing uncertainty by validating assumptions physicians may hold. We demonstrate the potential impact of using very large patient databases to answer a variety of questions clinicians may ask, as well as providing potential evaluation directions. Our provided case studies are meant to deepen the readers’ understanding of embedding model behavior. In each case study, the top ten most related disease conditions in the embedded space are retrieved, and their associations are discussed.

As a reminder, D2D is using only EHR records (list of diseases a patient was diagnosed) and no domain knowledge information. The model is then learning vector representation for each of the diseases such that *contextually similar* diseases are closer in the embedded space. Displayed use cases show that embedded space can be characterized as discovering conditions in phenotypes that are *i)* a similar condition (including same disease present on different organ), *ii)* different stages of the same condition, and *iii)* causative and/or effective conditions to central disease.

#### Case study 1: Chronic kidney disease Stage I (ICD-9 code: 585.1)

As an example, we show the nearest neighbors of *Chronic kidney disease Stage I* (CKD) in Table 2. The model was able to learn to accurately map within its closest proximity (given are values of Cosine similarity) successive stages of this chronic disease without including any domain knowledge. The recovery of disease stages was observed in other case studies, including high fatality diseases of acute myocardial infraction (ICD-9 code: 410.00) and lung cancer (ICD-9 code: 162.9).

#### Case study 2: Multiple Sclerosis (ICD-9 code: 340)

*Multiple sclerosis*, a chronic disease involving damage to the sheaths of nerve cells in the brain and spinal cord, is discussed next (Table 3). The reasons for this disease are not yet well understood, but the autoimmune process appears to be caused both by genetic and environmental factors -e.g., viral infections in early life^55^. Discovered associations in this case study support these statements. From the top 10 retrieved phenotypes, we observe that different inflammations of neural tissue (e.g., spinal cord, optical nerves, brain), late effects of neural tissue bacterial infections as well as late effects of nervous system injuries are highly ranked. A better understanding of these inflammations, bacterial infections, and physical injuries and their relation to multiple sclerosis may help address the heterogeneity found in patients, and also improve the treatment of the disease, including prevention in some cases. Moreover, high ranks of different scleroses (including notorious ALS disease) and spina bifida (a birth defect in which a baby’s spinal cord fails to develop properly) may strongly indicate that diseases in this phenotype are determined by the genes their carriers possess. The ranked list of genes identified by this study can be found in the Supplement.

#### Case Study 3: Septicemia (ICD-9 code: 995.91)

*Sepsis* (blood infection) is a condition caused by an overwhelming immune response to infection. From the left side of Table 4 we observe that “Severe sepsis” and “Septic shock” are discovered as the most related to the disease code, “Sepsis”, validating previously known relationships (given that these could be considered as stages of sepsis in general). More surprising neighboring disease codes include infections (both bacillus and non-bacillus, including fungi that easily penetrate into blood - candidiasis and mycoses) and inflammations on various body parts and organs. Additional high rank related diagnoses were hyperosmolality and hypernatremia which shows that the obtained retrievals are capable of detecting well known indicators of sepsis. Using this knowledge about related phenotypes may help physicians react earlier to potential septic cases and reduce mortality of the biggest killer disease in California (e.g., by reacting earlier to an infection that has not turned septic yet).

#### Case Study 4: Congestive Heart Failure (ICD-9 code: 428.0)

From the family of heart diseases we show disease-disease associations for one of the most deadly diagnoses. *Congestive Heart Failure* (CHF), a disease in which the heart becomes weaker over time (i.e., heart’s pumping power is weaker than normal) while usually expanding its volume. Discovered disease-disease associations for CHF (Table 4 right side) are dominated by conditions involving asynchronous heartbeat due to fibrillation, flutter, tachycardia and blockades of cardiac nerve that often cause these asynchronous heartbeat conditions. Longer periods of asynchronous heartbeat cause weakening of heart muscle due to irregular blood flow. Pulmonary disorders can lead to pulmonary hypertension which can result in heart failure. A similar mechanism can be caused by chronic kidney failure condition; however it is not present in the phenotype. The reason for such an oversight can be lack of chronic kidney disease instances in HCUP database, due to the fact that patients suffering from chronic kidney diseases are regular visitors to the hospitals (regular dialysis treatment) and are not considered inpatients. Thus, proposed models would not be able to learn proper vectors for such a condition, indicating a limitation of the study, but not a weakness of the proposed approach. Heart disorders of aortic valve and hearth cells are also present in the CHF phenotype, and represent indicators of scars on the heart, that are well correlated to the CHF. Similar traits, as in picking disease causes in the phenotype have been observed in three more high fatality diseases: pneumonia (ICD-9 code: 486), acute respiratory failure (ICD-9 code: 518.81) and renal failure (ICD-9 code: 586).

### DAG2D based disease-genes associations

In order to evaluate the potential power of the DAG2D model to identify gene-disease associations we conducted the following experiment. First, we randomly selected 20% of the diseases that have associated genetic data (the diseases found in the GWAS data set) and removed all their gene associations from the training data. Although we removed genetic information, these diseases are not removed from the original EHR data, so that we are able to learn their vector representations. Second, we learn DAG2D on the data set where the remaining 80% of diseases remained associated with their corresponding gene information data. The DAG2D model then contains low-dimensional representations of diseases and genes in the same embedding space, and we evaluated model performance by measuring how many of the removed genetic associations were correctly retrieved.

We compared DAG2D to modularity, spectral, and LDA methods, trained on 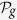 data. Graphs for modularity and spectral were constructed such that diseases and genes represent nodes where links between diseases were based on co-occurrence information, while links between genes and diseases were created based on disease-gene associations. Having learned disease and gene representations for each of the diseases from the test set we found the top *K* genes based on similarity in a low-dimensional space. In addition two trivial predictors are included: disease-gene co-occurrence, predicting genes that most commonly appeared in records and most frequent gene, always predicting the most commonly occurring genes.

Similar to the previous section, in Table 5 we report averaged *precision*@*K*, which is defined as a percentage of genes that are correctly identified out of top *K* retrieved. We find that the proposed DAG2D method outperforms the baselines for almost all values of *K*, except on the very challenging prediction of *K* = 1, where DAG2V was second best. Interestingly, LDA is the least accurate one in both DAG2D and D2D experiments, which can be explained by the fact that this method performs poorly on short “documents” (the average patient record in our data has only 7.62 diagnoses).

To delve deeper into the obtained results, the top results obtained in disease-gene association discovery is provided: we identified all 185 of 185 genes known to be associated via the GWAS to congestive heart failure (ICD-9 code: 428.0), 90/90 genes associated to hypothyroidism (ICD-9 code: 244.9), 108/111 for chronic airway obstruction (ICD-9 code: 496), and 100/111 for osteoarthrosis (ICD-9 code: 715.90).

The three genes found for chronic airway obstruction, not present in the 111 genes identified from GWAS studies, are SH3RF1, LOC645177 and SPAG16. While examining the literature, we found that all three genes have similar levels of gene expression in a number of tissues including the lungs. Additionally, proteomic assays reveal high expression in platelet blood cells for SH3RF1^56^, which are shown to influence chronic airway obstruction in the past^57^ and in bone marrow stem cells for SPAG16^58^.

As most diseases have no available genetic associations (i.e., gene-disease associations were not available from the EBI-NHGRI GWAS catalog), we find that discovered associated genes often have protein and/or microarray expression in an associated tissue or that there is a mechanism that can potentially explain certain non-obvious associations, which will hopefully be unraveled in the near future by genetic research. The full list of genes ranked according to the DAG2D model is provided in the Supplement for further examination and as a resource for future genetic research.

## Supplement

In addition, provided are two supplementary files: *disease-phenotype.csv* and *disease-genotype.csv*. Both files store query disease name and its ICD-9 code in first two columns, and the other columns include top 50 nearest diseases in phenotype, sorted, and top 1000 closest genes, sorted, obtained by the D2D and DAG2D models, respectively.

## Conclusion

We propose a novel model for phenotyping and gene discovery, building upon the latest advances in neural language models. The described approaches allow for unsupervised learning from patient records, as well as seamless incorporation of expert, domain knowledge into the learning process. The methods learn low-dimensional representations of diseases and genes in a common embedding space, setting the foundation for disease-disease and disease-gene relationship discovery through trivial K-nearest neighbor searches in the new vector space. The experiments on large-scale EHR data demonstrate that the proposed approaches significantly outperform the existing state-of-the-art methods on important tasks of phenotyping and gene discovery in the emerging area of computational phenotyping. Benefits of the approaches will advance clinical research and practice by accelerating our understanding of disease and gene associations.

## Additional Information

**How to cite this article**: Gligorijevic, D. *et al*. Large-Scale Discovery of Disease-Disease and Disease-Gene Associations. *Sci. Rep.* **6**, 32404; doi: 10.1038/srep32404 (2016).

## Supplementary Material

Supplementary Information

Supplementary Information

## Figures and Tables

**Figure 1:**
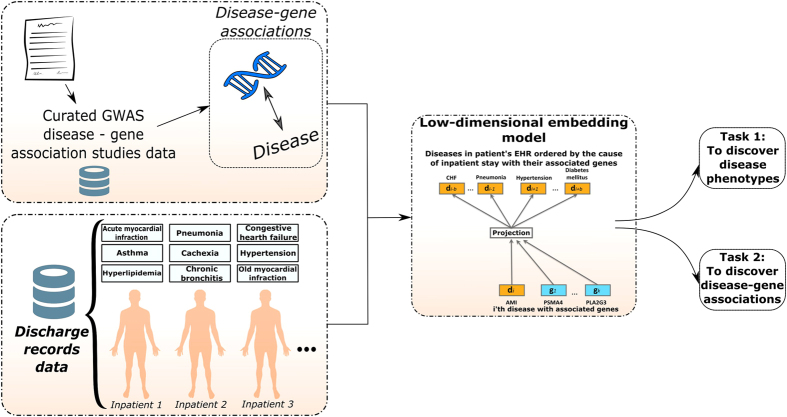
Graphical summary of the approach proposed in this study. Heterogeneous data obtained from large scale discharge records and hand curated disease-gene associations are used to jointly learn meaningful vector representations of disease and gene concepts in a latent vector space, where interactions of diseases and genes are retrieved and discovered.

**Table 1:** Precision and gene overlap for various competing models for the task of phenotype discovery, evaluated using disease-gene associations.

Data	*K*	Average precision*@K*				Average perc. of overlapping genes*@K*			
		1	2	5	10	1	2	5	10
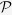	D2D	**0.9449**	**0.9367**	**0.9225**	**0.9047**	**0.8159**	**0.7966**	**0.7564**	**0.7111**
	Modularity (Adjacency)	0.8575	0.8457	0.8284	0.8130	0.5198	0.4893	0.4508	0.4145
	Spectral (Adjacency)	0.7181	0.7007	0.6795	0.6640	0.3052	0.2779	0.2311	0.2006
	Modularity (Comorbidity)	0.8493	0.8412	0.8110	0.7865	0.5586	0.5315	0.4681	0.4204
	Spectral (Comorbidity)	0.8375	0.8316	0.8190	0.7974	0.5288	0.4989	0.4520	0.3964
	Comorbidity graph	0.7268	0.7134	0.6915	0.7068	0.1582	0.1496	0.1465	0.1554
	Disease co-occurrence	0.5616	0.5516	0.5439	0.5668	0.1448	0.1329	0.1264	0.1261
	LDA	0.5260	0.5094	0.4913	0.4217	0.1040	0.0989	0.0864	0.0853
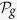	DAG2D	**0.9598**	**0.9444**	**0.9239**	**0.9079**	**0.8486**	**0.7963**	**0.7237**	**0.6720**
	Modularity (Adjacency)	0.8711	0.8604	0.8503	0.8389	0.5303	0.5082	0.4706	0.4340
	Spectral (Adjacency)	0.9165	0.9102	0.9020	0.8926	0.7524	0.7430	0.7277	0.7110
	Disease and genes co-occurrence	0.6978	0.6985	0.7093	0.7071	0.1058	0.1042	0.1018	0.0935
	LDA	0.5795	0.3874	0.3253	0.2831	0.1136	0.0781	0.0760	0.0652

**Table 2:** Four nearest disease neighbors for Chronic Kidney Disease Stage I.

Phenotype disease	Cosine Similarity
Chronic kidney disease Stage II (mild)	0.9361
Chronic kidney disease Stage III (moderate)	0.8652
Chronic kidney disease Stage IV (severe)	0.7647
Chronic kidney disease unspecified	0.6923

**Table 3:** Ten nearest disease neighbors of the Multiple Sclerosis phenotype retrieved by the D2D model.

Multiple Sclerosis
Late effect of spinal cord injury
Other causes of myelitis
Neuromyelitis optica
Acute infective polyneuritis
Late effects of intracranial abscess or pyogenic infection
Late effects of viral encephalitis
Acute (transverse) myelitis NOS
Amyotrophic lateral sclerosis
Spina bifida without mention of hydrocephalus unspec. region
Primary lateral sclerosis

**Table 4:** Ten nearest disease neighbors for the Sepsis and Congestive heart failure phenotypes retrieved by the D2D model.

Sepsis	Congestive heart failure unspecified
Severe sepsis	Other primary cardiomyopathies
Septic shock	Atrial fibrillation
Intestinal infection due to Clostridium difficile	Other specified forms of chronic ischemic heart disease
Candidiasis of other urogenital sites	Atrial flutter
Other and unspecified mycoses	Other chronic pulmonary heart diseases
Systemic inflammatory response syndrome	Paroxysmal ventricular tachycardia
Hyperosmolality and-or hypernatremia	Cardiac pacemaker
Pressure ulcer stage III	Aortic valve disorders
Proteus infection	Other left bundle branch block
Other shock without mention of trauma	Old myocardial infarction

**Table 5:** *precision@K* results for gene discovery.

*K*	1	2	5	10
DAG2V	0.6978	**0.8056**	**0.7293**	**0.6711**
Modularity	0.4760	0.4874	0.4902	0.4689
Spectral	**0.7803**	0.7551	0.6705	0.6387
LDA	0.2300	0.2570	0.3560	0.4180
Co-occurence	0.4691	0.3867	0.2998	0.2416
Most Frequent	0.2000	0.3467	0.4324	0.3887
